# Supplementary material for: Insights into Layered Oxide Cathodes for Rechargeable Batteries
Source: Molecules. 2021 May 26;26(11):3173. doi: 10.3390/molecules26113173 (PMC8198143; doi:10.3390/molecules26113173)
Supplement: Supplementary file 1 [file molecules-26-03173-s001.zip › molecules-1226541-supplementary.pdf]

Supplemental information for

Insights into Layered Oxide Cathodes for Rechargeable Batteries

Julia H. Yang [1], Haegyeom Kim [2] and Gerbrand Ceder [1,2,*]

[1] Department of Materials Science and Engineering, UC Berkeley, Berkeley, CA 94720, USA; juliayang@berkeley.edu
[2] Materials Sciences Division, Lawrence Berkeley National Laboratory, Berkeley, CA 94720, USA;
haegyumkim@lbl.gov
* Correspondence: gceder@berkeley.edu

Supplemental Information for calculating the DFT-SCAN band structure of $LiCoO_2$

The semilocal SCAN calculation is performed using the Vienna Ab Initio Simulation Package (VASP) [1,2] using the projector augmented wave (PAW) method [3,4] with a reciprocal space discretization of 25 K-points per $Å^{-1}$ and a plane wave energy cutoff of 520 eV. The calculation is converged in self-consistent calculation to 10^{-6} eV in total energy and 0.01 eV/Å on atomic forces to obtain the charge density.

A non-self-consistent calculation is then performed along the high-symmetry k-points for a rhombohedral lattice [5] and the resultant band structure is parsed using the electronic band structure module in pymatgen [6].

References

1. Kresse, G., & Furthmüller, J. Efficient iterative schemes for ab initio total-energy calculations using a plane-wave basis set. *Physical Review B* **1996**, *54*(16), 11169–11186. https://doi.org/10.1103/PhysRevB.54.11169

2. Kresse, G., & Furthmüller, J. (1996). Efficiency of ab-initio total energy calculations for metals and semiconductors using a plane-wave basis set. *Computational Materials Science* **1996**, 6(1), 15–50. https://doi.org/https://doi.org/10.1016/0927-0256(96)00008-0

3. Blöchl, P. E. Projector augmented-wave method. *Physical Review B* **1994**, 50(24), 17953–17979. https://doi.org/10.1103/PhysRevB.50.17953

4. Kresse, G., & Joubert, D. From ultrasoft pseudopotentials to the projector augmented-wave method. *Phys. Rev. B* **1999**, 59(3), 1758–1775. https://doi.org/10.1103/PhysRevB.59.1758

5. Setyawan, W., & Curtarolo, S. High-throughput electronic band structure calculations: Challenges and tools. *Computational Materials Science* **2010**, 49(2), 299–312. https://doi.org/https://doi.org/10.1016/j.commatsci.2010.05.010

6. Ong, S. P., Richards, W. D., Jain, A., Hautier, G., Kocher, M., Cholia, S., … Ceder, G. Python Materials Genomics (pymatgen): A robust, open-source python library for materials analysis. *Computational Materials Science* **2013**, 68, 314–319. https://doi.org/https://doi.org/10.1016/j.commatsci.2012.10.028
